# Dual targeting of CCR2 and CX3CR1 in an arterial injury model of vascular inflammation

**DOI:** 10.1186/1477-9560-8-14

**Published:** 2010-09-13

**Authors:** Maya R Jerath, Peng Liu, Mary Struthers, Julie A DeMartino, Roche Peng, Laurence B Peterson, Anne-Marie Cumiskey, Lihu Yang, Mauricio Rojas, Dhavalkumar D Patel, Alan M Fong

**Affiliations:** 1 Department of Medicine, University of North Carolina, Chapel Hill, NC, USA; 2 Merck & Co. Rahway, NJ, USA; 3 Novartis Institutes for Biomedical Research, Basel, Switzerland

## Abstract

**Objectives:**

The chemokine receptors CCR2 and CX3CR1 are important in the development of coronary artery disease. The purpose of this study is to analyze the effect of a novel CCR2 inhibitor in conjunction with CX3CR1 deletion on vascular inflammation.

**Methods:**

The novel CCR2 antagonist MRL-677 was characterized using an in vivo model of monocyte migration. To determine the relative roles of CCR2 and CX3CR1 in vascular remodeling, normal or CX3CR1 deficient mice were treated with MRL-677. After 14 days, the level of intimal hyperplasia in the artery was visualized by paraffin sectioning and histology of the hind limbs.

**Results:**

MRL-677 is a CCR2 antagonist that is effective in blocking macrophage trafficking in a peritoneal thioglycollate model. Intimal hyperplasia resulting from vascular injury was also assessed in mice. Based on the whole-blood potency of MRL-677, sufficient drug levels were maintained for the entire 14 day experimental period to afford good coverage of mCCR2 with MRL-677. Blocking CCR2 with MRL-677 resulted in a 56% decrease in the vascular injury response (n = 9, p < 0.05) in normal animals. Mice in which both CCR2 and CX3CR1 pathways were targeted (CX3CR1 KO mice given MRL-677) had an 88% decrease in the injury response (n = 6, p = 0.009).

**Conclusion:**

In this study we have shown that blocking CCR2 with a low molecular weight antagonist ameliorates the inflammatory response to vascular injury. The protective effect of CCR2 blockade is increased in the presence of CX3CR1 deficiency suggesting that CX3CR1 and CCR2 have non-redundant functions in the progression of vascular inflammation.

## Background

Atherosclerosis is an inflammatory process that is initiated by endothelial dysfunction leading to an increase in adhesiveness of platelets and lymphocytes to the injured region of the artery[1]. The inflammatory response continues with the migration of monocyte derived macrophages, dendritic cells, and a subset of T cells to the area. Finally, smooth muscle cell migration and proliferation occurs. Chemokines and their receptors play an important regulatory role for these processes and genetic studies have revealed specific polymorphisms that are associated with coronary artery disease and carotid artery occlusive disease in man[2,3]. Two chemokine receptors, CX3CR1 and CCR2, have been widely studied in mouse models of inflammatory disease. Each receptor has been shown to play a role in inflammatory cell trafficking. CX3CR1 is expressed on monocytes, natural killer cells, and a subset of T cells[4]. CX3CR1 is also found on smooth muscle cells[5]. Its ligand CX3CL1 (fractalkine) is a membrane bound chemokine that is increased in atherosclerosis[6]. We have shown that this molecular pair can mediate leukocyte adhesion and migration under physiological flow conditions *in vitro*[7]. In addition, using CX3CR1 deficient animals, we have shown that these mice have decreased macrophage and dendritic cell accumulation during vascular inflammation and are protected from intimal hyperplasia in an arterial injury model [8,9]. Similarly CCR2 is also expressed on the majority of blood monocytes, a subset of T cells, as well as other leukocytes. CCR2 is responsible for directed cell migration towards its primary ligand monocyte chemotactic protein 1 (MCP-1, CCL2) and can also respond to other chemokines of the MCP family [10]. Furthermore, CCR2 deficient animals exhibit a decreased susceptibility to atherosclerosis and also decreased intimal hyperplasia following arterial injury[11,12]. These two receptors are differentially expressed in monocyte subsets across species from rodent to man[13]. Recent results show that monocyte migration following kidney ischemia reperfusion injury is dependent on both CCR2 and CX3CR1[14]. We hypothesized that CCR2 and CX3CR1 have non-redundant effects in vascular inflammation and sought to determine their combined effect in a murine arterial injury model. Arterial injury induces vessel wall inflammation by stimulating platelet adherence, leukocyte recruitment, and vascular smooth muscle cell migration and proliferation. These same cellular responses are the basis of atherosclerosis and restenosis.

## Methods

### Monocyte recruitment

Defined pathogen-free female C57Bl/6 mice were purchased from Taconic Farms and housed in a sterile pathogen-free environment. Monocytes were recruited to the peritoneal cavity over the course of 3 days following a single i.p. injection of 1 ml sterile 10% Brewer's thioglycollate medium. Three days after the injection of thioglycollate, peritoneal exudate cells (PECs) were lavaged from the mice with two 5 ml washes of warm Dulbecco's PBS (without Ca^++ ^and Mg^++^) containing 5% fetal calf serum and 10 units/ml heparin. All groups contained 10 mice. Following lavage, PECs from each mouse were washed once with Dulbeccos PBS containing 5% fetal calf serum and the total number of viable cells was determined microscopically with trypan blue. FACS analysis was used to determine the total number of monocytes per mouse by staining with FITC anti F4/80. MRL-677 [patent: US7230008 B2 Appl. No. US2004923594A], or an equivalent volume (0.5 ml) of vehicle (DMSO:Cremophor:Saline, 2:4:94, v:v:v) were administered by oral gavage at the indicated doses once a day during the 3 day thioglycollate elicitation.

### Affinity of MRL-677 for CCR2 in whole blood

Germ free C57Bl/6 mice obtained from Taconic were used for the following experiments. Murine blood was collected by cardiac puncture into tubes containing EDTA to inhibit clotting. For each sample, blood from three to four animals was pooled together and diluted 1:1 with cold PBS containing 1 mM EDTA. MRL-677 (100 X concentration) in DMSO (50 μl) was added to 5 ml of blood to achieve the desired concentration and these samples incubated for 1 hour at RT with gentle rotation. PBMCs were rapidly isolated on Ficoll-Hypaque gradients (Lympholyte-Mammal Cedarlane laboratories Cat # CL5120). The buffy coat containing principally monocytes and lymphocytes (PBMCs) was isolated from the interface layer and transferred to a fresh tube. The medium was aspirated after centrifugation and 5 ml cold RBC lysis buffer (Sigma, Cat # R7757 155 mM ammonium chloride in 10 mM Tris-HCl buffer) was added and incubated for 5 min at RT to lyse residual red blood cells. Cold PBS (without Ca^++^/Mg^++^) was added to 10 ml, and cells harvested by centrifuging at 1200 rpm, for 6 min at 4°C. Cells were resuspended in approximately 0.8 ml cold binding buffer (50 mM HEPES, pH 7.2; 5 mM MgCl_2_; 1 mM CaCl_2_; 0.5% BSA and protease inhibitor cocktail (Sigma #P8340). An aliquot of these cells was retained to permit precise cell number determinations. ^125^I-murine MCP-1 (mMCP-1) binding to cells was then determined by standard filtration binding assays. Briefly 120 μl of binding buffer with 25 μl ^125^I-mouse MCP-1 (4 × 10^4 ^cpm, 40 pM) and 100 μl intact cells (500,000) were incubating for 1 hour at RT. The non-specific binding for each sample was determined by the inclusion of excess unlabeled mMCP-1 (200 nM) into an identical reaction mixture for 1 hour at RT.

### Murine vascular injury model

CX3CR1 knockout animals were backcrossed onto the C57Bl/6 background for 12 generations. Normal wildtype animals (Jackson laboratories) were used for controls. The femoral artery injury was performed using a procedure previously described[8]. Briefly, femoral arteries of 8-12 week old male mice in each experimental arm were injured by endoluminal passage of an angioplasty guidewire under general anesthesia. Mice were recovered and monitored after surgery. 14 days after injury, the mice were perfused with 4% paraformaldehyde for 20 minutes via cannulation of the left ventricle. The hind limbs were then harvested *en bloc*, fixed in paraformaldehyde and decalcified. Tissues containing the femoral artery were embedded in paraffin and cut into 5 mm sections for histologic and morphometric analysis.

In animals given MRL-677, the drug was administered by oral gavage at 30 mg/kg for the first two days of the protocol and thereafter in feed at an approximate dose of 15 mg/kg/day throughout the 14 days postoperatively. Blood levels of the antagonist were measured at times corresponding to peak (morning) and trough (evening).

### Histochemistry

Six to ten sections per femoral artery at 100 μm intervals were stained with hematoxylin and eosin. The sections from the area with maximal injury response were further evaluated by staining with the Combined Masson's elastin (CME) stain to visualize the arterial wall layers. The area of the vessel lumen, intima, and media were measured by computerized morphometry (Image J, NIH). Intimal hyperplasia was defined as the formation of a neointimal layer within the internal elastic lamina (IEL). Data were analyzed by t-test and p < 0.05 was considered significant.

### Immunohistochemistry

The serial sections embedded in paraffin were immunostained to detect the presence of monocytes using procedures previously described[8]. Briefly, after deparrafinization, the slides were treated with 0.3% hydrogen peroxide to inactivate endogenous peroxidase, washed in TBS, and antigen retrieval performed by incubating in a solution containing 0.1% CaCl_2 _and 0.1% trypsin for 30 minutes at 37C. After washing in TBS and blocking in 10% normal rabbit serum, the sections were incubated in a 1:250 dilution of F4/80 antibody (Serotec) overnight at 4C. The following day, the samples were washed in TBS and incubated in a rat anti mouse biotinylated secondary antibody (1:100 dilution) for 1 hour. After washing in TBS, the samples were incubated in streptaviden-horseradish peroxidase (Peroxidase Vectastain ABC kit, Vector Labs) for 1 hour at room temperature. The monocytes were then visualized using 3,3'diaminobenzadine (DAB, Sigma, St. Louis, Mo) as a substrate and the sections were counterstained with Gills solution.

## Results

We have previously shown that CX3CR1 knockout mice display a 58% decrease in intimal hyperplasia in a vascular injury model relative to wildtype animals [8]. In order to determine any relationship between the involvement of CX3CR1 and CCR2 in the inflammatory response in this model, we tested the effect of a specific CCR2 antagonist (MRL-677) in CX3CR1 knockout mice.

**Figure 1:**
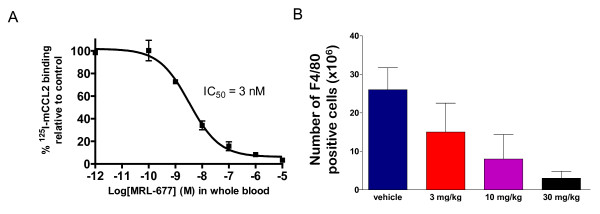
**A) MRL-677 added to murine whole blood at the concentrations indicated inhibited the binding of mCCR2 on PBMCs **. Each point represents the average of three measurements and the error bars represent the standard error of the mean. The potency (IC_50_) of the compound was determined to be 3.2 nM (+ 0.1 SE) and was determined by a four parameter fit of the data as shown. B) MRL-677 given orally inhibits recruitment of monocytes into the peritoneal cavity. The number of monocytes recruited to the peritoneal cavity was quantitated by F4/80 staining of the cellular exudate on day 3 post-intraperitoneal thioglycollate administration. Oral administration of MRL-677 for the three days at the doses indicated was able to inhibit monocyte recruitment to the peritoneal cavity.

MRL-677 has a murine whole blood potency (IC_50_) of 3.2 nM as determined by the ability to block ^125^I labeled-mCCL2 binding to peripheral blood mononuclear cells (PBMC) after *in vitro *whole blood treatment (Figure 1A). This is consistent with the potency of the compound to block mCCL2 binding to cells expressing recombinant mCCR2 (IC_50 _= 1.8 ± 0.4 nM), with a slight shift noted in whole blood, likely due to protein binding. MRL-677 was at least 100-fold more effective in inhibiting CCR2 compared to other chemokine receptors in standard competition binding experiments: murine CCR5 (selectivity >200 fold), human CCR1 (selectivity > 5000 fold), CCR3 (selectivity >500 fold), CCR8 (selectivity >500 fold), CXCR1 (selectivity >5000 fold), CXCR2 (selectivity >5000 fold), and CXCR3 (selectivity >500 fold). Table 1 summarizes the findings. All assays were performed as competition binding assays using radioactively labeled chemokine receptor ligands.

**Table 1:** CCR2 Antagonist MRL-677 Competition

	**IC**_**50 **_**or % inhibition**
Murine CCR2	1.8 ± 0.4 nM

Murine CCR5	418 ± 76 nM

Human CCR1	24% @ 10 μM

Human CCR3	No inhibition @ 1 μM

Human CCR8	No inhibition @ 1 μM

Human CXCR1	8% @ 10 μM

Human CXCR2	11% @ 1 μM

Human CXCR3	3% @ 1 μM

To confirm the activity of MRL-677 *in vivo*, we evaluated its effect of CCR2 blockade on monocyte recruitment in response to inflammation in C57Bl6 mice. The intraperitoneal administration of thioglycollate evoked a robust monocyte infiltration to the murine peritoneal cavity (26 ± 6 × 10^6 ^cells) compared to mice without application of thioglycollate (1 × 10^6 ^cells). Administration of MRL-677 at doses of 3, 10, or 30 mg/kg/day inhibited monocyte recruitment in the peritoneal cavity upon thioglycollate stimulation by approximately 49%, 75%, and 89% respectively (Figure 1B). Drug levels of MRL-677 assessed at trough for these experiments were below the limit of detection (<1 nM) 24 hours after 3 and 10 mg/kg/day doses and was 5 nM 24 hours after the highest dose tested at 30 mg/kg/day. Therefore, MRL-677 was administered by oral gavage at 30 mg/kg for the first two days and thereafter in feed at a dose of ~15 mg/kg/day in order to evaluate the effect of inhibiting CCR2 on vascular inflammation in response to injury. In addition to reducing animal handling and the stress that accompanies it, the administration of the drug in feed was anticipated to lead to higher trough drug levels to ensure complete blockade.

In the vascular injury experiments, we first measured the blood concentration of MRL-677 everyday for the complete experimental course of 14 days in both wildtype and CX3CR1 KO mice. We achieved an average trough level of 0.43 ± 0.11 μM for wildtype mice and 0.68 ± 0.41 μM for CX3CR1 KO mice. This indicates that sustained therapeutic drug concentrations considerably greater than those anticipated to give >95% coverage of CCR2 based on the mouse whole blood potency (whole blood IC_95 _= 0.06 μM, corresponding to 19 fold the IC_50_) were achieved during the 14 day treatment.

**Figure 2:**
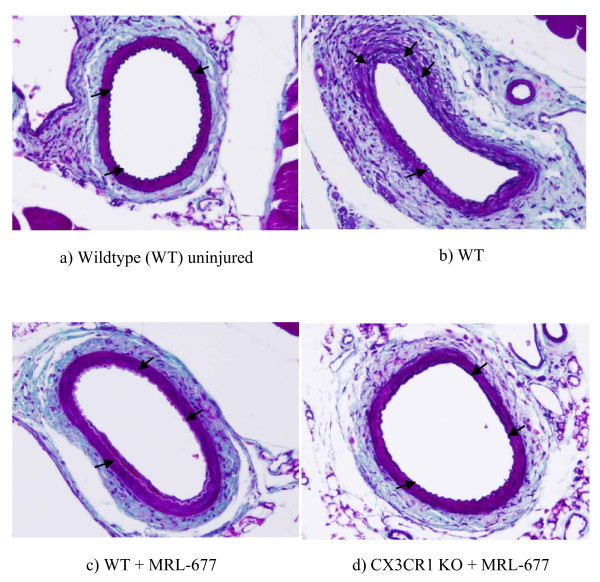
**CME stained cross section of mouse femoral artery (a) with no injury and (b)14 days after endoluminal injury in wildtype mouse, (c) wildtype mouse fed CCR2 antagonist, and (d) CX3CR1 KO mouse fed CCR2 antagonist **. IEL is marked by arrows. Note the lack of a visible intima with the IEL defining the vessel lumen in the uninjured artery (a). The neointima overlies the IEL in the injured animals (b-d).

**Figure 3:**
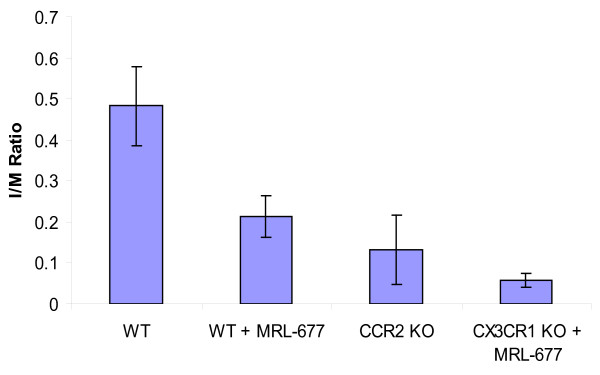
**Shown are the average intimal/media ratios for each experimental arm **. Graph depicts the mean ± standard error in each group. Statistical significance was achieved for differences between WT and WT+MRL-677, as well as between WT+MRL-677 and CX3CR1 KO+MRL-677.

**Figure 4:**
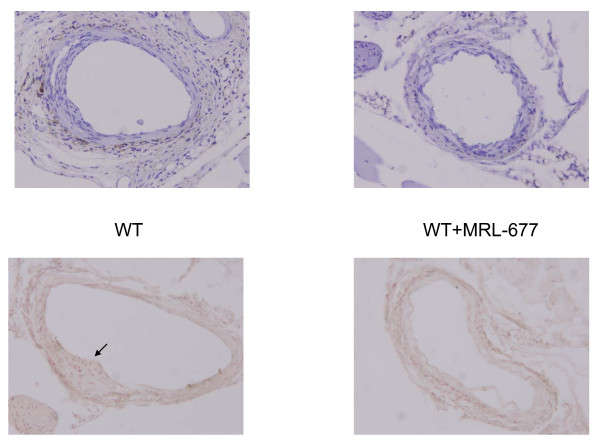
**Monocytes within the femoral artery were detected by immunohistochemistry using the F4/80 antibody **. A representative section from wildtype animals and those treated with MRL-677 is shown. The top pair of pictures was counterstained in Gills solution.

Then we examined the intimal hyperplasia by measuring the ratio of the area of the intima over the media (I/M ratio) 14 days after the femoral artery injury procedure. Treatment of wildtype mice with the CCR2 antagonist resulted in 56% reduction in intimal hyperplasia compared to wildtype mice without antagonist treatment (0.482 ± 0.097 vs. 0.213 ± 0.05, p < 0.05) (Figure 2). In addition, the degree of protection from intimal hyperplasia observed in the normal mice fed the drug is similar to that seen in CCR2 deficient mice (0.213 ± 0.05 vs. 0.133 ± 0.084, p = NS). Furthermore, there was a dramatic reduction in vascular injury observed in the CX3CR1 KO animals given the CCR2 antagonist (I/M ratio of 0.056 ± 0.017) in which there was an 88% decrease in the development of intimal hyperplasia relative to untreated wildtype animals. Thus, knocking out the CX3CR1 pathway together with blocking CCR2 function via MRL-677 significantly decreased the intimal hyperplasia and this decrease is greater than inhibiting CCR2 alone (p = 0.009, Figure 3). In order to determine whether or not monocyte trafficking played a role in the change in intimal hyperplasia observed, we performed immunohistochemistry on sections. Figure 4 shows that while monocytes are seen in injured femoral arteries from wildtype mice, there is a significant decrease in monocyte numbers in MRL-677 treated animals. These data suggest that although CX3CR1 and CCR2 can both influence vascular inflammation and remodeling, their roles are non-redundant and additive.

## Discussion

Both CX3CR1 and CCR2 are important in the development of coronary artery disease [15]. These receptors may play a role in the migration of monocytes and dendritic cells to the atherosclerotic plaque as well as in the proliferation of vascular smooth muscle cells observed during the vascular remodeling stage. In this study, we show that the compound MRL-677 is a potent and effective antagonist for murine CCR2. In a thioglycollate induced assay of monocyte migration in mice, MRL-677 nearly completely inhibits F4/80^+ ^monocyte migration at a dose of 30 mg/kg (given as oral gavage) which gave trough drug levels of 5 nM. However, significant inhibition of monocyte recruitment was also observed at doses of 3 and 10 mg/kg during which drug levels fell below the IC_50 _of the compound. Administration of the drug at approximately 15 mg/kg/day in feed resulted in increased plasma drug levels at trough (PM) with drug levels reaching average levels over 400 nM. This is likely due to the fact that the drug is ingested over a prolonged period of time which blunts the peak-to-trough relative to a single oral gavage.

We have also shown that blocking CCR2 with this low molecular weight antagonist ameliorates the inflammatory response to vascular injury. This finding is consistent with previous results using a CCR2 antibody in a rhesus model of vascular injury[16]. Therefore targeting of CCR2 may be a good therapy for reduction of restenosis after angioplasty. However, simultaneous blockade of the CCR2 and CX3CR1 signaling pathways has an even greater effect than blockade of either receptor alone. We have previously published that CX3CR1 deficient mice exhibited a 58% decrease in intimal hyperplasia compared to wildtype animals (p = .0017)[8]. Among CCR2 antagonist treated animals, the CX3CR1 KO mouse exhibited a significantly lesser degree of intimal hyperplasia on drug compared to the wildtype animals given the drug. This result suggests a complementary and perhaps synergistic effect on the development of the vascular inflammatory response of these two chemokine pathways. Our findings support reports published recently using atherogenic ApoE -/- mice that were deficient in both CCR2 and CX3CL1 [17,18]. They show that there is reduced macrophage accumulation and atherosclerotic lesion formation in these animals compared to the single knockout mice, suggesting that CX3CR1 and CCR2 can act independently to promote monocyte recruitment to the atherosclerotic lesions. It has been suggested that CCR2 may be important for the migration of monocytes to the atherosclerotic plaque while CX3CR1 may play a larger role in the retention of the monocytes to the lesion[19].

Therefore, our results further imply that CCR2 and CX3CR1 have independent, non-redundant roles in vascular inflammation and the later stages of coronary artery disease. Approaches to target both may have more beneficial effects than targeting each one separately.

## Conclusion

The results of our study indicate that CCR2 and CX3CR1 act by distinct and non-redundant mechanisms to mediate vascular inflammation following arterial injury.

## Competing interests

MS, LP, AC, JD, RP and LY declare that they are current or former employees of Merck and Company. DP declares that while his involvement in the study was during his employment at the University of North Carolina, he is currently an employee of the Novartis Institutes for Biomedical Research.

## Authors' contributions

MJ performed the studies and data analysis, PL aided in the studies and analysis (vascular injury model). LP, AC, and RP performed studies and data analysis (monocyte recruitment and whole blood affinity of MRL-677). LY synthesized and provided MRL-677. MS participated in the study design, JD participated in the study design, MR performed the surgeries, DP participated in the study design, and AF performed some experiments and wrote the manuscript. All authors read and approved the final manuscript.
